# Editorial: The role of mortalin in biology and disease

**DOI:** 10.3389/fcell.2023.1196430

**Published:** 2023-04-11

**Authors:** Jong-In Park

**Affiliations:** Department of Biochemistry, Medical College of Wisconsin, Milwaukee, WI, United States

**Keywords:** molecular chaperone, mortalin, inhibitor, cancer, neurodegeneration, viral infection, anemia, HSP70

The heat shock protein (HSP) 70 family chaperones are central regulators of many cellular processes requiring protein quality control. Mortalin (also called mtHSP70, GRP75, PBP74, and HSPA9) is a member of this family initially identified as the mitochondrial HSP70 (Craig et al., 1989; Leustek et al., 1989). While the critical roles of mortalin in different mitochondrial processes are well established, growing evidence suggests that mortalin also has previously unexpected roles outside mitochondria. In line with this, disease-associated mortalin alterations and their pathophysiological significance have been noted. This Research Topic aimed to update recent advances in mortalin functions in different pathophysiological contexts and the potential therapeutic molecules that target mortalin and its protein network.

Increasing evidence suggests the significance of mortalin in cancer as its correlation with patient outcomes are often detected in different tumors. Esfahanian et al. review the literature to cover this information and also analyze the RNAseq datasets from the Cancer Genome Atlas (TCGA) database to demonstrate this correlation in different cancers. Esfahanian et al. show that the prognostic impact of mortalin, determined by its RNA levels, can differ between tumors and discuss the factors limiting RNA expression data interpretation. Upregulated mortalin expression confers a survival advantage to different types of tumor cells, rationalizing its candidacy as a therapeutic target. Yoon et al. and Elwakeel introduce a brief history of mortalin beginning from its identification as an anti-senescence factor in cells (Wadhwa et al., 1993), which granted the name “mortalin,” and review the identification of the tumor suppressor TP53 as a key mortalin interaction partner. Notably, while nuclear localization of TP53 is essential for its tumor-suppressive effects, mortalin can inhibit TP53 nuclear localization by sequestering the tumor suppressor in the cytoplasm. This is a widely known mechanism underlying the pro-tumorigenic effects of mortalin. Elwakeel comprehensively reviews the mortalin-TP53 interaction and the rationale for targeting this physical interaction for tumor suppression. Mortalin functions require various interaction partners, such as co-chaperones, regulators, and clients in different subcellular compartments. Esfahanian et al. review these effectors and regulators of mortalin for mitochondrial and ER homeostasis, trafficking of macromolecules, proteostasis, signal transduction, and complement activation. Esfahanian et al. also review how post-translational modifications such as phosphorylation, acetylation, oxidation, and ubiquitination affect mortalin function and interactions with its binding partners with a significant focus on UBXN2A-mediated mortalin ubiquitination and degradation.

Yoon et al. and Elwakeel review different chemical inhibitors that can abrogate the mortalin-TP53 interaction in cancer. Renu Wadhwa’s group has made a notable drug discovery effort in this regard. They screened a chemical library of 12,000 compounds to discover three novel triazole derivatives that shift mortalin distribution from perinuclear to pan-cytoplasmic regions and induce TP53 nuclear translocation and reactivation. Meidinna et al. briefly review the first two chemicals, Mortaparib and Mortaparib^Plus^, and report their latest discovery of the third compound Mortaparib^Mild^. Interestingly, all these compounds disrupt the mortalin-TP53 interaction and simultaneously inhibit PARP1, similar to Olaparib, a clinically used PARP1 inhibitor. So, one may attribute the tumor suppressive effects of the Mortaparib series compounds partly to PARP1 inhibition.

In contrast to Mortaparib, there is also an inhibitor developed without the original intention of mortalin targeting. Sulfur heteroarotinoid A2 (SHetA2) is an investigational cancer drug currently evaluated in a clinical trial. Benbrook describes her group’s initial focus on designing synthetic versions of retinoic acid and the subsequent discovery of the derivatives that work independently of retinoic acid receptors. Surprisingly, SHetA2 suppressed tumor cell survival through unexpected interactions with HSP70 protein complexes, including GRP78, HSC70, and mortalin. Benbrook proposes that all these chaperones are targets of SHetA2 while attributing its tumor suppressive effects substantially to mitochondrial damages caused by disrupted mortalin protein networks. Benbrook also discusses the first-in-human Phase-I clinical trial that tests SHetA2 capsules in patients with advanced or recurrent ovarian, cervical, and endometrial cancer (NCT04928508) as well as potential drug combination strategies.

The significance of mortalin is also suggested in other diseases. A critical etiological factor for neurodegeneration is defective proteostasis, which grants a role for mortalin in this disease. Priyanka and Seth review mortalin alterations in Alzheimer’s, Parkinson’s, and human HIV-1-associated neurocognitive disorders cases. Priyanka and Seth discuss how these pathophysiological conditions affect mortalin networks and functions for mitochondrial homeostasis and neuronal toxicity prevention. Priyanka and Seth also discuss the role of mortalin in the extracellular release of HIV-1 negative regulatory factor and subsequent neuronal death. While not often detected in cancer, mortalin mutations are associated with neurodegeneration and congenital sideroblastic anemias (CSA). CSA is a group of rare genetic disorders characterized by abnormal iron accumulation in erythrocyte precursors. While Priyanka and Seth review mortalin mutations associated with Parkinson’s disease, Vishwanathan and D’Silva characterize different loss-of-function mortalin variants identified from CSA patients. Vishwanathan and D’Silva show that CSA-associated mortalin mutations lead to mitochondrial dysfunction in yeast, a model convenient for an initial characterization of mortalin mutations given the high homology between human and yeast HSP70 genes. Their results provide valuable insight into the molecular mechanisms involved in the pathophysiology of CSA.

There are also areas wherein mortalin involvement is immerging. Esfahanian et al. briefly review the role of mortalin in facilitating oncogenic effects of the Raf/MEK/ERK, PI3K/AKT, Wnt/GSK3/β-catenin signaling pathways. Data are increasing lately in this field. For example, although not covered in this Research Topic, mortalin can determine cellular steady-state MEK1/2 activity by regulating MEK1/2-PP1α interaction (Wu et al., 2017). Mortalin also suppresses lethal mitochondrial stress arising upon oncogenic KRAS- or BRAF-induced MEK/ERK activation (Wu et al., 2020a; Wu et al., 2020b). Notably, this mortalin dependency may be exploitable to suppress MEK/ERK-deregulated tumor cells resistant to MEK/ERK-targeting drugs (Wu et al., 2021). It is crucial to address how mortalin regulates the two other signaling pathways. Extracellular mortalin and its interaction with the tumor microenvironment is another area that needs attention (Jubran et al., 2017; Rozenberg et al., 2018). Lastly, developing a chemical probe more specific to mortalin and other HSP70 paralogs is important. While not covered in this Research Topic, the effort to advance MKT-077 has led to the development of JG-98 derivatives, which have improved selectivity (Shao et al., 2018; Shao et al., 2022). These newer areas will require updates in a future Research Topic.
